# Dental students in an orthodontic course flipped classroom: A semi‐experimental study on knowledge, practice, attitude, and satisfaction

**DOI:** 10.1002/cre2.868

**Published:** 2024-03-03

**Authors:** Maryam Karandish, Zahra Karimian, Mina Parastar

**Affiliations:** ^1^ Department of Orthodontics, School of Dentistry Shiraz University of Medical Sciences Shiraz Iran; ^2^ Department of e‐Learning in Medical Sciences, Virtual School and Center of Excellence in e‐Learning Shiraz University of Medical Sciences Shiraz Iran; ^3^ Student Research Center, Dental School Shiraz University of Medical Sciences Shiraz Iran

**Keywords:** blended learning, dental education, flipped classroom, online learning

## Abstract

**Objective:**

The purpose of this study was to determine the students' attitudes before and after the flipped classroom, and the effectiveness of this method to promote the students' lateral cephalograms tracing abilities, students' satisfaction and their final exam scores.

**Materials and Methods:**

This is a single‐group quasi‐experimental research conducted on dental students of Shiraz University of Medical Sciences (SUMS), Iran in 2019. The intervention was carried out in a blended learning approach with the flipped classroom model. Thirty‐five fourth‐year dental students participated in a flipped classroom held during a semester for the lateral cephalograms tracing course. The students were provided with the educational materials before the class time through multimedia learning tools and the class time was devoted to discussions. The students were asked to fill out four questionnaires (pretest/posttest attitudes, pretest/posttest self‐assessments of theoretical knowledge and practical skills in cephalograms, posttest of satisfaction from quality of the course, and posttest of students' views about effectiveness of blended learning tools) and final exam scores of students.

**Results:**

Students' attitudes toward e‐Learning were improved after the flipped classroom and the quality of this method was acceptable to the students (*p* < .001). Their self‐assessment of theoretical knowledge and practical skills were promoted (*p* < .001). While all blended learning tools averaged more than the cut‐off‐point, short lecture (5.11 ± 0.98) and live feedback (4.98 ± 1.07) were considered to be the most efficient interactive tools.

**Conclusion:**

It seems that the flipped classroom has a positive effect on increasing students' knowledge, attitude, and satisfaction. In general, this method of learning seems to be favored by dental students.

**Clinical Significance:**

The findings showed that blended learning had a positive effect on increasing knowledge, performance, and satisfaction among dental students. Therefore, blended learning as a reliable method might be used in training dental students.

## 1 INTRODUCTION

Dentistry is a combination of art and science among medical sciences that includes a set of theoretical, practical, and clinical courses. The students, as well, have to strengthen various cognitive, psycho‐motor, and emotional dimensions of their skills (de Azevedo et al., 2018; de Azevedo Rde et al., 2015; Kellesarian, 2018). So, dental students should practice, drill the concepts, and interactively discuss the subject in the classroom (Eachempati et al., 2016). While teachers often try to convey and transfer the knowledge and curriculum content in the traditional way (Brown & Manogue, 2001; Golden, 1989). This problem is often challenged by teachers engaged in medical sciences due to the large, highly specialized, and diverse content of the course (Golafrooz Shahri & Khaghanizade, 2010; Nowrozi et al., 2011). Although the traditional lecture‐based method is useful in conveying the content of the course, it probably reduces interaction between teacher and student (Matheson, 2008; Snell, 1999).

The behaviorist approaches with an emphasis on content transfer are not compatible with nowadays learning context and students prefer methods based on problem‐solving, critical thinking, and interpersonal interactions and communications (Blouin et al., 2009; Eachempati et al., 2016; Karandish, 2020). It should also be noted that students have different individual capabilities and learning styles, and traditional education cannot fulfill this variety of learning styles (Alcota et al., 2011; Smolen et al., 2016). Fortunately, students are familiar with and fully engaged with new technologies and welcome technology‐based education (Eachempati et al., 2016; Faraone et al., 2013; Maresca et al., 2014; Qutieshat et al., 2020). A new approach to learning is called blended learning. Blended learning is actually combining the positive points of traditional face‐to‐face methods with new online methods providing a more effective environment for students to learn (Maresca et al., 2014). Various models of blended learning are used in education (Tolks et al., 2016). They include concepts/methods like rotation (Station Rotation, Lab Rotation, Individual Rotation, and Flipped Classroom), self‐blend, flex, and enriched virtual model (Heydari et al., 2019). Numerous studies have confirmed the effectiveness of these methods (Nijakowski et al., 2021; Pahinis et al., 2007; Reissmann et al., 2015; Ullah et al., 2021; Varthis & Anderson, 2018). One of the most widely used models of blended learning is the flipped classroom. The flipped classroom also known as reverse, inverse, or backwards classroom is a type of blended learning. In this method, the students learn the basic knowledge before class time via websites, videos, podcasts, texts, and articles. Afterward, during face‐to‐face teaching, case‐based and problem‐based discussions occur under the guidance of the teacher (Lee & Kim, 2018; Persky & McLaughlin, 2017; Tucker, 2012). This method might help the students to have a better understanding of the subject, retain the learned knowledge, and be able to use it in real situations (Eachempati et al., 2016; Lee & Kim, 2018; Persky & McLaughlin, 2017).

Some studies on flipped learning demonstrated improvements in students' cognitive skills such as problem‐solving and students' satisfaction for enabling them to learn at their own pace and use personalized learning techniques (Khanova, Roth, et al., 2015; Sait et al., 2017; Veeramani et al., 2015). The flipped classroom was found to be more effective for teaching periodontal diagnosis and treatment planning, compared to the traditional lectures (Lee & Kim, 2018). It also appeared to engage students to a greater degree in a pediatric dentistry course than the traditional lectures (Gadbury‐Amyot et al., 2017). Nishigawa et al. (2017) reported higher efficiency of the flipped classroom and team‐based learning than the traditional styles in fixed prosthodontics education. Bohaty et al. (2016) flipped a pediatric dentistry course and reported a higher number of students with A grades than the previous year when the course was taught in a traditional way through lectures. The flipped classroom combined with the case‐based learning achieved better results for endodontic residents' training and resulted in the residents' higher satisfaction (Sun et al., 2018). However, the effectiveness of the flipped classroom in health sciences education is still debated because of the conflicting results. Whillier and Lystad (2015) found that the flipped classroom did not improve students' performance nor satisfaction in learning neuroanatomy, suggesting that it is not a suitable approach for learning heavy and complex content. Khanova, McLaughlin, et al. (2015) reported negative results of the flipped classroom for the students who are more familiar with the traditional lectures and Ihm et al. (2017) suggested that the design of the flipped classroom must be based on the students' educational needs and the factors that affect students' learning outcomes. Kim et al. (2018) recommended considering the students' learning styles and personality types to design a more effective flipped learning classroom. Also the limitations of the flipped classroom such as the reduced contact with the teacher and the misunderstandings of the material delivered before the class should be considered. In addition, there should be motivators to increase the success of the flipped classroom because of its challenging nature (Sait et al., 2017). As a result, there needs to be more research and evidence to confirm the effectiveness of the flipped classroom in different aspects of dental education. In recognition of the importance of the learner‐centered approaches in dental education, and to investigate the effectiveness of flipped classrooms in orthodontics education, a lateral cephalogram tracing course was flipped for the fourth‐year dental students at Shiraz University of Medical Sciences (SUMS). The aim of this study was to evaluate the:

1) Students' attitudes toward e‐Learning before and after the course.
2) Students' satisfaction with the quality of flipped classroom dimensions.
3) Students' opinions about the effectiveness of each interactive blended learning tools.
4) Students' self‐assessments of their theoretical knowledge and practical skills.
5) Students' knowledge based on their final exam score.

## 2 METHODS AND MATERIALS

### 2.1 Participants

All 35 fourth‐year dental students at the Dental School of SUMS participated in a flipped classroom held for an orthodontic course teaching lateral cephalometric radiograph principles and tracing.

### 2.2 Study design

This is a single‐group quasi‐experimental study on dental students. Educational intervention and data collection were performed from February 2018 to July 2019. The course was presented in a blended learning approach with a flipped classroom model. First, the students were provided with the materials (e‐Contents) before the face‐to‐face classroom, within a Learning Management System (LMS) platform prepared in SUMS. Students were able to study at home at their own pace and control the number of contents they viewed. In this research, variety of face‐to‐face and e‐Learning tools were used. Multimedia content was provided to the students before the course and the students had to read them before attending the respective class. In the face‐to‐face session, the instructor first presented a summary of the topic (a short lecture of about 30 min). Then, according to the case‐based discussion (CBD), clinical cases were presented with images, face‐to‐face discussions were held around a clinical problem, and students actively participated in the discussion. CBDs were held to reinforce the students' comprehension of the cephalogram tracing concept. During the question and answer (Q&A) session, the teacher provided live feedback to the students and clarified any misunderstandings and misconceptions by reviewing the most important points of the lesson in the classroom. In the classroom, there were also quizzes at the end of each topic. In addition, part of the homework was done at home through the forum of LMS (presenting dental cases with clinical images through asynchronous discussion). The online forum was used to hold discussions during the course, addressing students' questions regarding *principles of lateral cephalograms tracing*, *finding the landmarks*, and *interpreting the lines and the angles* theoretically. and also tracing the lateral cephalograms practically. To remember and perpetuate the e‐contents presented, objective self‐examination (e‐quiz) was presented in each content with interactive feedback. The effectiveness of the course based on Kirkpatrick's model was investigated on two levels of reaction and learning (Falletta, 1998). Kirkpatrick's model is one of the most common models in the evaluation of training programs, which evaluates the effectiveness of training courses in four levels: reaction, learning, behavior, and result. Since the duration of the course was one semester, it was not possible to evaluate levels 3 and 4, that is, permanent and long‐term changes.

### 2.3 Educational content

The production and evaluation of multimedia was carried out in accordance with the standards of the Center of Excellence in E‐Learning affiliated with SUMS. All the topics in theoretical and practical sections were developed in the form of interactive multimedia with storyline software (SCORM version 2004). The situation and time of studying, reading, and downloading of files was tracked through the LMS. The podcasts (the instructor's recorded voices) of the sessions were provided to the students. It was possible to download e‐content for students.

### 2.4 Research tools

The research tools consisted of five researcher‐made questionnaires (Q1−Q5).

#### 2.4.1 Assessment of reaction (feeling)

Q1. Quality: The first questionnaire concerned the quality of the course and consisted of 18 questions on a Likert scale (from 6 = strongly agree to 1 = strongly disagree) and the cut‐off‐point was 3.5. The cut‐off‐point means the minimum acceptable value. In Likert scale questionnaires, to calculate the cut‐off‐point value of 50%, the total score of the options is divided by the number of options. In this research, to calculate the cut‐off‐point value, the total score of options (1 + 2 + 3 + 4 + 5 + 6) is divided by the number of options (6), the answer is 3.5. this questionnaire consisted of three components: course organization (six items), active learning (eight items), and flexibility (four items).

Q2. Effectiveness of blended learning components: The second questionnaire comprised eight questions on a Likert scale (five options from 5 = very high to 1 = very low), concerning the students' evaluations of the effectiveness of blended learning components/tools that were used in this method and generally analyzed their views toward receiving the course through the flipped classroom. The cut‐off‐point was 3 (the total score of options [1 + 2 + 3 + 4 + 5] is divided by the number of options [5], the cut‐off‐point is 3).

Q3. Attitude about e‐Learning: The third questionnaire consisted of 19 questions dealing with the students' attitudes toward e‐Learning before and after taking the course, on a Likert scale (across six grades from 1 = strongly disagree to 6 = strongly agree) and the cut‐off‐point was 3.5.

([1 + 2 + 3 + 4 + 5 + 6] is divided by the number of options [6], the cut‐off‐point is 3.5). The components of the attitude questionnaire included: attitude toward e‐Learning in general (five items), perception toward the use of e‐Learning (11 items), and perception of the consequences of e‐Learning (five items).

#### 2.4.2 Assessment of learning

Q4. Self‐assessments of knowledge: The fourth questionnaire investigated the students' self‐assessments of their theoretical knowledge and practical skills before and after the flipped classroom on a six‐point scale) from 6 = very high to 1 = very low (and comprised two parts; the theoretical part consisting of “principles of lateral cephalograms tracing,” “finding the landmarks,” and “interpreting the lines and the angles.” The practical part consisted of “tracing the cephalograms,” “finding the landmarks,” and “drawing the lines and the angles correctly.”

Q5. Final exam score: According to the routine of all educational courses, at the end of the academic semester, the final exam was held in the form of essay questions (based on Bloom's taxonomy at the level of application, analysis, and evaluation). The final exam was conducted in person. The test score was between 0 and 20 and the minimum passing score was 12. The number of questions, the type of questions, and the test time were the same for all students.

Since there isn't a control or comparison group in this research, the students' knowledge scores were compared with their similar group in the semester (October to January 2018), just before the flipped group who completed the course in the traditional method (only face‐to‐face).

#### 2.4.3 Other questions

In addition, demographic information (gender and age) and the level of the students' computer skills were added (consisted of six questions evaluating the students' computer skills on a Likert scale [six options from 6 = very high to 1 = very low]). The cut‐off‐point was 3.5.

Also, free comments of the students regarding the good and bad points of the flipped classroom from their points of view were also collected with the questionnaire.

**Figure 1:**
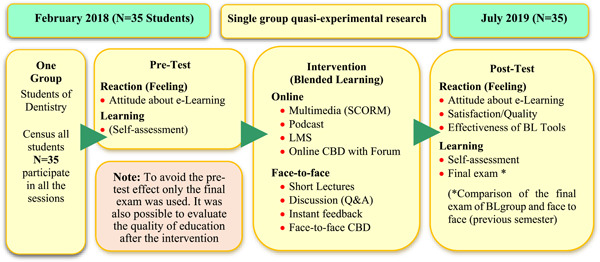
A schematic view of the research design of educational intervention.

A schematic view of the research design is shown in Figure 1.

#### 2.4.4 Validity and reliability of research tools

To determine the content validity of the questionnaires, content validity index (CVI) calculations were used by reviewing the opinions of 10 specialists in medical education and orthodontic fields. The acceptable level of agreement was 79% (Waltz & Bausell, 1981). To measure the reliability of the questionnaires, after collecting 40 questionnaires the internal consistency with Cronbach's *⍺* index was evaluated.

Validity: The opinions of 10 experts were used to measure the face and content validity of the questions. After the grammatical corrections of four questions from the attitude questionnaire and three questions from the satisfaction/quality questionnaire, all questions were confirmed with a validity of more than 80%.

Reliability: The reliability of the attitude questionnaire with 19 questions was 90%, and the measured reliability of the subcomponents including the students' self‐perceptions toward e‐Learning, the application of e‐Learning, and the consequence of e‐Learning were 78%, 76%, and 85%, respectively. The overall reliability of the questionnaire regarding the satisfaction/quality of the course which had 18 items was 94% and the calculated reliability of the subcomponents including the course organization, the active learning, and the flexibility of the course was 91%, 88%, and 84%, respectively. The reliability of the questionnaire with eight questions about the effectiveness of the blended learning tools was 85%. A summary of the characteristics of the research tools is given in Table 1.

**Table 1:** The characteristics of each of the researcher‐made tools.

Kirkpatrick's levels	Questionnaires	Evaluation	Number of items	Scale	Cut‐of‐point
Reaction (feeling)	Satisfaction/quality	Posttest	18	1−6	3.5
	Effectiveness of blended learning tools	Posttest	8	1−5	3
	Attitude about e‐Learning	Pretest/posttest	19	1−6	3.5
Learning (changes)	Self‐assessment of knowledge	Pretest/posttest	7	1−6	3.5
	Students' knowledge (final exam's score)	Posttest	12	0−20	12

### 2.5 Data analysis and statistical tests

To determine the means of students' opinions regarding the quality of the course (satisfaction) and the effectiveness of interactive blended learning tools, a one‐sample *t*‐test was used. To compare the students' scores in the final exam with the scores of the same‐level students in the previous semester, an independent *t*‐test was used. To compare students' attitudes before and after the intervention, as well as self‐assessment of students on theoretical and practical subjects before and after educational intervention, a paired sample *t*‐test was used. Furthermore, to compare students' opinions based on background variables, independent samples *t*‐test and ANOVA were utilized. Statistical analysis was performed using SPSS 24 statistics and the *p*‐values less than .05 were considered significant in this study.

### 2.6 Ethics statement

Students completed the questionnaires with awareness and satisfaction. They were assured that their comments did not affect their final score and therefore the questionnaires were analyzed anonymously. The current study was approved by the ethics committee, Shiraz University of Medical Sciences Review Board (No: IR.SUMS.REC.1399.028). This study was carried out at Shiraz University of Medical Sciences (SUMS) in Shiraz/Iran and we confirm that all experiments and methods were performed in accordance with relevant guidelines and regulations of the vice chancellor for Research, in SUMS. Written ethical approval was taken from the Shiraz University of Medical Sciences' local ethics committee approval number (IR.SUMS.REC.1399.028), and written informed consent obtained from all the participants.

## 3 RESULTS

### 3.1 Descriptive finding

**Figure 2:**
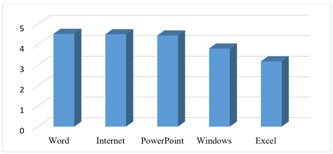
The average score students' computer skills from their perspective (cut‐off‐point = 3.5).

This study was conducted among 35 fourth‐year, undergraduate dental students in the School of Dentistry, SUMS, Iran. There were 24 female and 11 male students who participated in the study and the students aged between 21 and 26 years (students' mean age was 22.4 years). Five of the participants were married, 16 of the students resided in the dormitory, 15 of the students were living with their families, and 4 of them were living independently. None of the students were employed. In the present study, none of the students had previous experience in e‐Learning. The results showed that the students' computer knowledge in the fields of Windows, the Internet, and Microsoft Office programs including Word and PowerPoint was significantly high. Their knowledge of Excel was lower than the previous fields, but the difference was not significant (Figure 2).

### 3.2 Analytical finding

#### 3.2.1 Reaction

Students' reaction or feeling was investigated on two levels: satisfaction with the quality of the course and satisfaction with the effectiveness of blended learning tools.

##### Quality of the course

The results of the students' satisfaction about the quality of the flipped classroom dimensions represented favorable total mean scores and also favorable mean scores in all three subcomponents. The average of each item is shown in Table 2. The highest mean scores were associated with the course organization and the flexibility of the course (Table 3).

**Table 2:** The average score students' viewpoints about the items of quality of the course.

Components	Items	Mean	SD
Course organization	1. The quality of the e‐content was good	5.45	0.58
	2. E‐content were presented in order and sequence	5.28	0.71
	3. The e‐content covered the educational objectives	5.15	0.83
	4. Related examples were given in the classroom	5.15	0.75
	5. The course content was provided with appropriate quality	4.85	1.08
Active learning	6. Using multimedia (images, text, sound) was effective in my learning	4.94	0.98
	7. There was opportunity for discussion, questions, and answers in class	4.57	1.31
	8. My ability to discuss, critical reasoning, and thinking has increased	4.55	1.17
	9. I feel I have learned the course well	4.70	1.02
	10. Students participated in active questioning and learning	4.32	1.36
	11. There was opportunity to interact with the teacher in class	4.47	1.24
	12. I did not worry about taking notes and notes and focused on the lesson	4.83	1.20
	13. I learned from the questions‐answers and discussions of my classmates	4.43	1.31
	14. This teaching method motivated me to learn cephalometry	4.40	1.36
Flexibility	15. I read the e‐content and prepared for class before the session	4.40	1.31
	16. I had the opportunity to practice and repeat the content after class	4.66	1.08
	17. E‐content allows me to read according to the speed of my learning	4.70	1.08
	18. E‐content was compatible with our different learning styles	4.62	1.32

Abbreviation: SD, standard deviation.

Cut‐off‐point = 3.5.

**Table 3:** The average score students' viewpoints about the quality components of the course.

Components	Mean	SD	*t*	*p* Value
The course organization	5.13	0.69	11.26	<.001
Active learning	4.53	0.97	3.74	<.001
Flexibility	4.59	1.01	4.02	<.001
Total	4.75	0.81	10.51	<.001

Abbreviation: SD, standard deviation.

Cut‐off‐point = 3.5.

##### Opinions about the effectiveness of the blended learning tools

**Figure 3:**
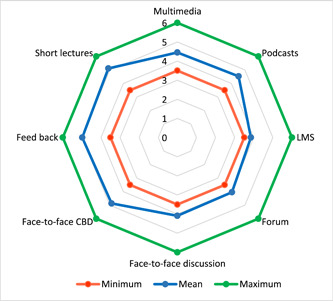
Students' opinions of the effectiveness of learning tools in the flipped classroom.

According to the average opinions of the students, the following factors were the most effective tools in blended learning: the teacher's short lecture to review the lesson briefly (M = 5.11 ± 0.98), interactive Q&A or rapid feedback between the teacher and the students (M = 4.98 ± 1.07), face‐to‐face CBD (M = 4.87 ± 1.03), the teacher's podcasts (M = 4.53 ± 1.12), interactive multimedia (M = 4.45 ± 1.26), discussion in forum (M = 4.04 ± 1.33), and doing homework in LMS (M = 3.85 ± 1.30) (*p* < .001) (Figure 3).

##### Attitude

Comparing the students' attitudes toward e‐Learning before and after the blended course, improvements in the students' attitudes were observed in all items and these improvements were significant in both the total mean score and the mean scores of the three subcomponents (*p* < .001) (Table 4). There was no significant difference in the students' attitudes toward e‐Learning by gender. The residential status of the students did not make any significant differences in the mean values of the students' attitudes or the other items (*p* > .05).

**Table 4:** Comparison of students' attitudes about e‐Learning before and after the intervention.

Components	Groups	Mean	SD	*t*	*p* Value
Attitude toward the nature of e‐Learning	Pre	3.27	1.03	4.634	<.001
	Post	4.22	1.13		
Attitude toward the use of e‐Learning	Pre	3.08	0.73	5.981	<.001
	Post	3.88	0.94		
Attitude toward the consequence of e‐Learning	Pre	3.52	0.87	5.946	<.001
	Post	4.20	0.93		
Total mean score	Pre	3.25	0.74	6.289	<.001
	Post	4.06	0.84		

Abbreviation: SD, standard deviation.

Cut‐off‐point = 3.5.

#### 3.2.2 Learning

##### Students' self‐assessments

All students passed the final exam. In addition to the final exam, students expressed their knowledge before and after about the theoretical and practical subjects of the course in the form of self‐evaluation (it is worth mentioning that in the comparison group [in person/the previous semester], the number of male students = 14, and the number of females = 20). The results of learning of students including self‐assessments and final exam scores are shown in Table 5.

**Table 5:** Comparison of self‐assessments and final exam scores of students.

Components		Groups	Mean	SD	*t*	*p* Value
Students' self‐assessments of their knowledge and practical skills (cut‐off‐point: 3.5)						
Theory	Principles of lateral cephalograms	Pre	1.66	1.04	15.38	<.001
		Post	4.62	1.19		
	Principles of lateral cephalograms tracing	Pre	1.49	1.04	13.94	<.001
		Post	4.68	1.23		
	Finding the landmarks	Pre	1.96	1.28	14.29	<.001
		Post	4.81	1.09		
	Interpreting the lines and the angles	Pre	1.55	1.05	13.33	<.001
		Post	4.57	1.33		
Practice	Tracing correctly	Pre	1.47	1.01	6.851	<.001
		Post	4.85	1.04		
	Finding the landmarks correctly	Pre	1.77	1.20	14.85	<.001
		Post	4.74	0.98		
	Drawing the lines and the angles correctly	Pre	1.74	1.25	13.82	<.001
		Post	4.38	1.11		
Comparing the final exam score of students with their similar group in the previous semester (cut‐off‐point: 12)						
Final exam score	*N* = 35, score between (14.5−18.75)	Blended learning	16.94	1.10	3.691	<.001
	*N* = 34, score between (14.5−17.75)	Face‐to‐face	16.02	0.96		

Abbreviation: SD, standard deviation.

The students' self‐assessments showed significant improvements in their theoretical knowledge and their practical skills after the blended course (*p* < .001).

##### Knowledge score

The students' knowledge score was compared with their similar group in the previous semester (only face‐to‐face training). The results showed that at a significant level, the average scores of students who were trained in the blended learning method were higher than those who were trained in the traditional (*p* < .001) (Table 5).

### 3.3 Results extracted from free comments

An open‐ended question was included at the end of each questionnaire for free comments and the students recorded their opinions and feelings. The students clearly expressed a good feeling about this learning method. Some example quotes include:

- When I'm sure I have the e‐content before the course starts, I'm more focused in face‐to‐face discussions and don't worry about handouts.
- Multimedia provides more interesting and rich content.
- I can repeat the more difficult parts of the lesson several times.
- Providing a summary of the main parts of the lesson as a brief lecture is very helpful.
- For me, living in a dormitory with other students, it is very useful to have the electronic content before the start of the course. Because I can study it at the right time and place.

## 4 DISCUSSION

Improvements in technology have resulted in the introduction of new learning approaches like blended learning. Practitioners in the dental field require the ability of utilizing theoretical knowledge in practice and the ability of linking the newly learned materials to what they already know. Blended learning such as the flipped approach that provides active engagement of students might be beneficial to deepen the students' understanding of the learning content. In this research, the effectiveness of blended learning was investigated with the flipped classroom approach on dental students in five fields.

### 4.1 Attitude

The findings from the first research question showed that the blended learning experience in students had a positive effect on their views about the nature, application, and future consequences of e‐Learning. It was in accordance with the results of a meta‐analysis conducted by Hew and Lo (2018) who reported students' positive attitudes toward the flipped classroom. In addition to its necessity and applicability in medical science education, the attitude toward electronic learning is also influenced by the generational characteristics of students. Most of today's students were born in the digital age and are natives of the virtual world, and therefore have more affinity with virtual education. Also, Kohli et al. examined first‐year dental students' short‐term and long‐term knowledge retention in different learning environments, used the TRIL questionnaire to investigate students' perceptions toward these methods (conventional, flipped, and spaced learning), and found that although long‐term knowledge retention is higher in the traditional method than the flipped and the spaced learning, the flipped method had the lowest decrease in long term retention of the information which might be due to the availability of the contents that the students were provided with. In addition, the students had positive opinions of this method and were eager to receive more courses through this approach (Kohli et al., 2019). In the current study, the students' enthusiasm in such classes was observed. One possible explanation for this positive attitude, which was offered by Giuliano and Moser (2016), is the students' easy access to the materials presented before class time. Suner et al. (2019) who investigated the dental students' opinions toward mobile learning suggested that the application of instructional aids such as visual tools in teaching dentistry increases students learning and this might be another reason for students' positive opinions.

### 4.2 Satisfaction/quality

Based on the findings of the present research, three components of *course organization*, *active learning*, and *flexibility* had a higher score than the cut‐off‐point which actually showed a high degree of satisfaction with the quality of education.

Basically, in courses where the amount of course content is large, the professors have to manage the class in a lecture style, and therefore there is no opportunity for interaction in the classroom, but the flipped classroom approach makes students receive and study the course content before the course begins. Instead, they should do homework and discuss and exchange ideas in the classroom. Moraros et al. and Park et al. applied flipped method for the students of public health and reported their positive feedback. They showed that the flexibility of this method which fulfills the students' need for competence and active learning might be an explanation for this positive response (Moraros et al., 2015; Park & Howell, 2015). The course organization such as good scientific contents, the appropriate sequence of the materials, in‐class teaching plans adjusted based on the students' educational needs, and reasonable combination of images, texts, and voices were other positive points that made this method acceptable for the students in this study. The combination of multimedia, podcasts, text, and images in this study also reached the same results as Moraros et al. and Park et al. This was similar to the results represented by Ha et al. (2019) who showed that to ensure the students' implementation of this method, the material presented before the class must be attractive and related to in‐class presented material, and the teacher's acquaintance with this method affects the outcomes of this approach. Students have different learning styles and speeds (Van Horn et al., 2014). Multimedia, by combining images, text, sound, and electronic interactions between content and students, provides the basis for strengthening individual learning beyond the limitations of space and time. On the other hand, due to the fact that the e‐contents are available to the students before the class, they are not worried about taking notes, they are more focused in the classroom, and they can engage in active and collaborative learning in the classroom.

### 4.3 Knowledge and Practice

Also, the changes caused by the educational intervention were determined through self‐assessment and final exam score. The students' self‐assessment of theoretical knowledge, and practical skills of lateral cephalogram tracing were improved after the course and they had a better understanding of the lesson. Kohli et al. (2019) showed that comprehensive knowledge of the learned material was better, too. This might be because of easier concentration in the classroom due to the pre‐class acquaintance with the materials and the possibility of critical thinking and having interactive discussions in the environment that this method provides.

In addition, the final exam scores of students in a flipped classroom were compared to the scores of the students in the previous semester who were trained in the traditional method. The results showed that the flipped method was more effective. This result is in line with the research of Gallardo et al. (2022), Wang et al. (2021), and Özcan (2022), who compared the final scores of dental students in the flipped classroom and face‐to‐face training (Gallardo et al., 2022; Özcan, 2022; Wang et al., 2021).

Effectiveness of blended learning components: In this research, blended learning was presented with a flipped classroom approach and a combination of e‐tools (LMS, multimedia, podcasts, forum) and face‐to‐face training (short lectures, feedback, face‐to‐face CBD, and face‐to‐face discussion). Students found it difficult to work with LMS and the forums. The reason for this may be that in face‐to‐face discussions in the classroom, the feedback is quick and lively, but in the forum, there is often a long delay between questions, answers, and feedback. Also, in e‐tools such as forums, and LMS the presence of the teacher and live communication is less felt.

Among different learning tools exerted in this study, CBDs under the guidance of the teacher and active relationships with the teacher were considered the most efficient learning tools. Moazami et al. also found a higher willingness of fifth‐year dental students to participate in conventional methods of learning rotary instrumentation of canals rather than using technology and suggested that the students' fear of technology might be the reason for this preference (Moazami et al., 2014). Ha et al. also found that a positive teacher‐student relationship leads to higher students' motivation in the learning process. The instructor's lack of motivational skills to clarify the beneficial aspects of this approach and the instructor's lack of essential policies to convince students that they can learn through the flipped method had negative effects on the outcomes of this approach (Ha et al., 2019). Deprey reported that physical therapy students who experienced the flipped approach showed higher scores in the examinations; whereas those subjected to the partially flipped classrooms did not show the same results because they did not have the opportunity for practical application of the newly learned knowledge. They concluded that what promotes students' performances is the employment of the knowledge (Deprey, 2018). In the present study, the students realized that the CBDs were the most efficient and helpful in learning cephalometric analysis.

### 4.4 Background variables

No significant differences were observed in the mean scores of the students' attitudes between males and females. Men had higher scores only in one item which was associated with the application of the flipped approach in medical education. Suner et al. (2019) found that male students are more comfortable and more familiar with the technology. This might be the reason for male students' optimism regarding the future application of this method.

In line with the present research, Bohaty et al. (2016) demonstrated a promotion in a student‐centered approach and self‐directed learning by flipping a pediatric course. This was ascertained by a current study on the dental students' orthodontic topic. On the other hand, in spite of immediate improvement in individual learning and performance in a dental anatomy course in a flipped class, no long‐term difference was seen between flipped and traditional lectures (Chutinan et al., 2018). At the end of the course evaluation, the results of the current study showed positive feedback from students. Borit and Stangvaltaite‐Mouhat (2020) designed a game‐based flipped classroom and reached more joy experienced by the students. This study showed improvement in the students' attitude toward the flipped classroom as well. Due to the worldwide shift to full online classes because of Covid‐19 pandemic situation, it seems beneficial to compare the results of the flipped approach and the full online classes in these courses. Although the current research has confirmed the effectiveness of the blended learning approach, some research studies have also shown different results. Jensen et al. stated that only the use of face‐to‐face or electronic methods does not guarantee its effectiveness, but in fact, it was the amount of interaction between the teachers and the students that made a combined course effective (Jensen et al., 2015). In the research of Khojasteh et al. (2023) the results showed that the most important determining factor was the effectiveness of interactions between teachers and students.

It is suggested that comparative research be conducted to determine the effectiveness of e‐Learning methods and models. Also, random sampling increases the validity of the research. This research has been done only on dental students and can be done in other fields as well.

## 5 CONCLUSION

A blended learning approach with a flipped classroom model has a positive effect on increasing students' knowledge, attitude, and satisfaction and can be used as a selective approach in education. The flipped classroom model ensures that the student has access to the course content and increases the student's focus on interactions and questions and answers in face‐to‐face teaching. Also, access to e‐content makes it possible for students to study at their own time and place. Despite the importance of e‐content and the combination of e‐Learning and face‐to‐face methods, students are more inclined to live interactive methods. Face‐to‐face interactive methods are more effective due to fast feedback and no delay in receiving feedback as well as learning from peers. Also, the feeling of the teacher's presence in the classroom is one of the factors that seem to be important from the students' point of view. In general, it can be said that blended models are more effective in better education of cephalometric analysis in dental students by using the strengths of face‐to‐face and electronic methods.

## 6 LIMITATION

Only one group of dental students was admitted each year. Therefore, it was not possible to choose a comparison or control group. Also, this study was conducted before the Corona pandemic, when students did not have much experience in e‐Learning and their computer skills were weak. Given that students and teachers experienced deep e‐Learning during the COVID‐19 pandemic, and their skills improved, it is possible that the results of future research may differ from the present study. It should also be mentioned that the assessment tools were a few questionnaires and it may have limitations to provide a comprehensive and objective presentation of the teaching and learning process.

## AUTHOR CONTRIBUTIONS

Maryam Karandish devised the study concept and involved in study planning, performed educational intervention, collected the data and created the first and final draft of the study. Zahra Karimian devised the study concept and involved in study planning, developed the questionnaire, ran the reliability and validity tests and analyzed and interpreted statistical data, and revised the manuscript critically. Mina Parastar created the first draft of the study, helped editing the final version of the manuscript, contributed to study design and oversaw. All authors read and approved the final manuscript.

## CONFLICT OF INTEREST STATEMENT

The authors declare no conflict of interest.

## Data Availability

The data sets used and/or analyzed during the current study are available from the corresponding author on reasonable request.
